# Risk Factors for Thyroid Dysfunction among Type 2 Diabetic Patients in a Highly Diabetes Mellitus Prevalent Society

**DOI:** 10.1155/2013/417920

**Published:** 2013-12-23

**Authors:** Metab Al-Geffari, Najlaa A. Ahmad, Ahmad H. Al-Sharqawi, Amira M. Youssef, Dhekra AlNaqeb, Khalid Al-Rubeaan

**Affiliations:** ^1^Family and Community Medicine Department, Qassim University, P.O. Box 143, Buraidah 51411, Saudi Arabia; ^2^Biostatistics Department, University Diabetes Center, King Saud University, P.O. Box 245, Riyadh 11411, Saudi Arabia; ^3^Registry Department, University Diabetes Center, King Saud University, P.O. Box 245, Riyadh 11411, Saudi Arabia; ^4^Research Department, University Diabetes Center, King Saud University, P.O. Box 245, Riyadh 11411, Saudi Arabia; ^5^University Diabetes Center, King Saud University, P.O. Box 18397, Riyadh 11415, Saudi Arabia

## Abstract

Diabetes and thyroid dysfunction found to exist simultaneously. In this regard, the present study looked into the prevalence of different forms of thyroid dysfunction and their risk factors among Type 2 diabetic Saudi patients. *Methodology*. A cross-sectional retrospective randomized hospital-based study of 411 Type 2 diabetic Saudi patients of >25 years of age was conducted to test the prevalence of different types of thyroid dysfunction and their risk factors. *Results*. The prevalence of different types of thyroid dysfunction is 28.5%, of which 25.3% had hypothyroidism, where 15.3%, 9.5%, and 0.5% are clinical, subclinical, and overt hypothyroidism, respectively. The prevalence of hyperthyroidism is 3.2%, of which subclinical cases accounted for 2.7% and overt hyperthyroidism accounted for 0.5%. Risk factors for thyroid dysfunction among Saudi Type 2 diabetic patients are family history of thyroid disease, female gender, and duration of diabetes of >10 years, while the risk was not significant in patients with history of goiter and patients aged >60 years. Smoking and parity show a nonsignificant reduced risk. *Conclusion*. Thyroid dysfunction is highly prevalent among Saudi Type 2 diabetic patients, and the most significant risk factors are family history of thyroid disease, female gender, and >10 years duration of diabetes.

## 1. Introduction

Diabetes mellitus and thyroid dysfunction are the most common endocrine diseases seen in the adult population [1], while insulin or thyroid hormones metabolism can result in functional abnormalities of one another. The strong link between diabetes and thyroid diseases encouraged the American Diabetes Association (ADA) to propose that people with diabetes must be checked periodically for thyroid dysfunction [2]. Thyroid disease should be screened annually in diabetic patients to detect asymptomatic thyroid dysfunction [3]. At the same time, patients with thyroid dysfunction may need to be tested for the possibility of abnormal glucose metabolism, since excessive thyroid hormones cause increased glucose production in the liver, rapid absorption of glucose through the intestine, and increased insulin resistance [4]. The thyroid gland is one of the endocrinal systems of the human body and can be affected by sustained hyperglycemia and the continuous endeavors by the body to correct for this carbohydrate imbalance. Studies have shown that diabetes and thyroid dysfunction can be found to exist together where thyroid disease can affect glucose metabolism and the untreated thyroid dysfunction can affect the management of diabetes [3, 5]. The association of the two endocrinal dysfunctions has been reported in different societies throughout the last two decades [6–8]. Diabetic patients have susceptibility to different types of thyroid dysfunction, whether hypothyroidism or hyperthyroidism; at the same time, patients with thyroid dysfunction are susceptible to suffer from either Type 1 diabetes or Type 2 diabetes [1, 9].

Thyroid disorder is divided into clinical and subclinical disease, according to the hormonal levels and clinical presentation that will affect the follow-up and management plan. Thyroid dysfunction has been found to be more prevalent among diabetic population when compared with the normal population [5].

In Scotland, the prevalence of thyroid dysfunction was 13.4% among diabetics, reaching 31.4% in Type 1 female diabetic patients and falling to 6.9% in Type 2 male diabetic patients [6], while among Type 2 diabetic patients in Jordan, the overall prevalence of thyroid dysfunction was found to be 12.5% [8]. Subclinical hypothyroidism prevalence is variable among different ethnic groups or genders and was found to range from 4.8 to 6.3% [6, 10]. This was clearly shown in the United States, where prevalence was 5.8% in white women and 1.2% in black women but 3.4% in white men and 1.8% in black men [11].

Hyperthyroidism is a less common thyroid dysfunction in both general and diabetic patients. It has been reported to be 0.53% in Caucasian children with Type 1 diabetes mellitus [12] and 4.4% in Type 2 diabetic adult patients [13], while subclinical hyperthyroidism is reported to be approximately 2% [14].

There are many risk factors known to be associated with thyroid dysfunction in the general population, including age, gender, BMI, family history of thyroid disease, smoking, and pregnancy. Incidence of hyperthyroidism and hypothyroidism increases with age, especially beyond 20 years, and it has been established that female gender is 10–20 times more likely to have this medical problem than males [15]. Morbidly obese individuals show a high prevalence of overt and subclinical hypothyroidism, accounting for 19.5% [16]. The United Kingdom DNA collection for Graves' disease and Hashimoto's thyroiditis study identified family history of thyroid disease to be risk for thyroid dysfunction [17].

Smoking has been reported to be a risk for thyroid dysfunction, where higher T4 levels and lower TSH levels were reported among smokers but not among nonsmokers or former smokers. This may be explained by the toxicological effect of smoking on increasing levels of thyroxin binding globulin among smokers [18].

Estrogen has been shown to be associated with low risk for thyroid dysfunction, while pregnancy has a higher risk for developing hyperthyroidism [19].

Risk factors for thyroid dysfunction among diabetic patients are similar to what have been reported in nondiabetics, although they will vary with the type of thyroid dysfunction. Autoimmune thyroid disease is seen to be more frequent in the younger age group and females [20], while hypothyroidism among diabetic patients is more prevalent among women [21] and the older population [22]. Diabetes duration has been found to be a risk for thyroid autoimmune disuses in children and adolescents with type 1 diabetes [23], but it was not a risk in patients suffering from Type 2 diabetes in different ethnic groups [10, 24]. Goiter has been recognized as a risk factor for thyroid dysfunction in diabetic patients [10], as observed in nondiabetics [25]. Parity has been recognized to be a risk factor for thyroid dysfunction in diabetic women [26], which is also the case in nondiabetic mothers [27].

Saudi Arabia is the seventh of the top ten countries in terms of the prevalence of diabetes among the adult population aged 20–79, according to the IDF diabetes atlas 2012 [28]. The prevalence of thyroid dysfunction among Saudi diabetic patients was reported to be 16%, as opposed to 7% in nondiabetics, as shown by Akbar et al. in 2006 [7]. Since then, no study has been undertaken to investigate the relationship between diabetes and thyroid dysfunction, or to examine their risk factors in a community with high diabetes prevalence.

Since most studies investigating the prevalence of thyroid disease in diabetic patients have focused on Type 1 diabetes, the aim of this study is to assess the prevalence of different forms of thyroid dysfunction among Type 2 diabetic Saudi patients receiving care from April to October in 2012 at the University Diabetes Center (UDC) in King Abdul Aziz University Hospital (KAUH) in Riyadh. Determining the risk factors of thyroid dysfunction among Type 2 diabetic Saudi patients is part of this study's objectives.

## 2. Methodology 

This study is a cross-sectional retrospective randomized hospital-based study, in which 411 Type 2 diabetic Saudi patients were enrolled during the period April to October in 2012. The UDC is a tertiary diabetes center, which provides care for diabetic patients in Riyadh, the capital of Saudi Arabia.

Subjects recruited for this study were Saudi nationals with Type 2 diabetes of more than 25 years of age. The diagnosis of Type 2 diabetes was based on their initial presentation, using the American Diabetes Association (ADA) Criteria [29]. Inclusion criteria included adult Saudi Type 2 diabetic patients older than 25 years visiting the UDC during the study period. Exclusion criteria included patients who had previous thyroid surgery, pregnant women, Type 1 diabetes mellitus, and patients on the following medications: cordarone “anti-arrhythmic medication” lithium, interferon, iodide, or high doses of glucocorticoids.

Chart review was conducted to collect data, including demographic parameters that is, age, gender, and duration of diabetes, in addition to anthropometric measurements including weight, height, and body mass index (BMI) in addition to blood pressure that was collected from their last visit. Family history of diabetes or thyroid disease with or without goiter was reported, in addition to smoking history and parity for females. The presence of any associated diseases like hypertension, dyslipidemia, and thyroid disease including goiter was also documented. Laboratory data were collected from the patients' chart of the last visit, including HbA1c, fasting blood sugar (FBG), and 2 hour postprandial (2hpp) glucose, in addition to lipids profile including total cholesterol, triglyceride, high density lipoprotein (HDL), and low density lipoprotein (LDL). Thyroid function tests, namely, thyroid-stimulating hormone (TSH), free thyroxine (FT4) and free thyroxine (FT3), were collected during the same visit.

Each patient is evaluated for the presence of thyroid dysfunction, defined as biochemical abnormalities for clinical and subclinical hypothyroidism and hyperthyroidism, if they had been diagnosed and treated with either hypothyroidism or hyperthyroidism. Patients were classified as having clinical hypothyroidism if they have been diagnosed before and on thyroxin replacement therapy. Patients were labeled with sub-clinical hypothyroidism if they have TSH > 5 mIU/L but normal T4 (10.55–25.74 pmol/L), while overt hypothyroidism when TSH > 5.0 mIU/L with low T4 <10.55 pmol/L. Patients were labeled with hyperthyroidism if they have been treated surgically or given radioactive iodine therapy or on antithyroid medications. Diagnosis of subclinical hyperthyroidism is when TSH < 0.5 mIU/L with normal T4 (10.55–25.74 pmol/L), while overt hyperthyroidism is when TSH < 0.5 mIU/L with high T4 >25.74 pmol/L [30, 31].

## 3. Statistical Analysis

Data were entered into SPSS software version 17.0. Continuous variables were expressed as mean ± standard deviation, and categorical variables were expressed, as percentages. *t*-test was used for continuous variables and chi square test for categorical variables. Relative risk with 95% confidence interval (CI) was used to assess different risk factors of thyroid dysfunction among Type 2 diabetic patients. *P* value of less than 0.05 was used as a level of significance, and GraphPad software was used to plot different relative risk factors.

## 4. Results

Thyroid dysfunctions were found in 117 patients (28.5%) of the total sample of 411 Type 2 DM Saudi patients. The patients' baseline characteristics of the total sample showed a mean age of 59.0 ± 10.8 years but 59.3 ± 9.9 and 58.9 ± 11.3 for diabetic subjects with and without thyroid dysfunction, respectively, which is not significantly different (*P* = 0.732). Female gender percentage in the total sample was 52.3% but was significantly higher in patients with thyroid dysfunction of 68.6% when compared with normal thyroid subjects, where females accounted for 46.6% with *P* value < 0.0001. The mean diabetes duration was also significantly higher in patients with thyroid dysfunction than in normal ones (17.3 ± 9.0 versus 15.1 ± 8.6, resp.) with *P* value = 0.032. The percentage of patients with a positive family history of thyroid disease was significantly higher in patients with thyroid dysfunction (14.7%) versus (1.03%) among the normal thyroid diabetic patients with *P* value < 0.0001, while the percentage of family history of diabetes was not statistically different between the two groups. The percentage of smoking habits in the two groups did not show any significant difference.

The mean weight is 80.1 ± 15.4 and BMI of 31.5 ± 6.1 for the total sample, but without a significant difference between the patients with or without thyroid dysfunction. The height is significantly lower in patients with thyroid dysfunction, compared with the normal thyroid patients (157.3 ± 8.2 versus 161.2 ± 9.5, resp.) with *P* value < 0.0001. The mean systolic and diastolic blood pressures for the selected patients are 134.8 ± 16.5 and 74.1 ± 9.6, respectively, but did not show significant difference in patients with or without thyroid dysfunction. Goiter was found in 2.8% of the studied samples and in 4.8% in patients with thyroid dysfunction but 2.1% in patients without thyroid dysfunction, although it was not significant. The mean HbA1c, FBS, triglyceride, total cholesterol, HDL, and LDL for total sample were 8.5 ± 1.6, 8.7 ± 2.9, 1.5 ± 0.78, 4.2 ± 0.78, 1.2 ± 0.33, and 2.3 ± 0.69, respectively, but there was no significant difference for patients with or without thyroid dysfunction.

Thyroid function tests showed a significantly higher mean TSH value for patients with thyroid dysfunction than normal ones (4.7 ± 4.2 versus 2.6 ± 1.2, resp.; *P* value < 0.0001) and FT4 (16.6 ± 4.34 versus 15.6 ± 2.7 with *P* value 0.048), but no significant difference for FT3 (4.5 ± 2.03 versus 4.7 ± 0.84, resp., with *P* value 0.552) as shown in Table 1.

**Figure 1:**
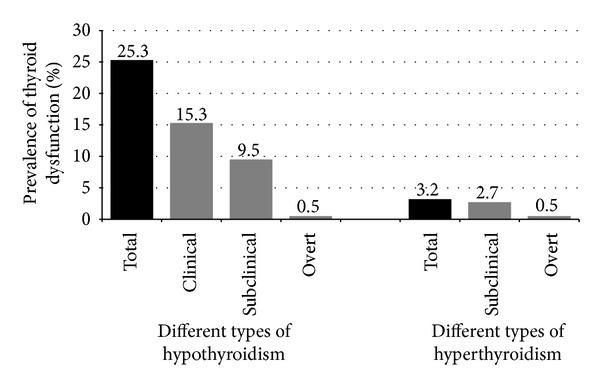
Prevalence of different types of thyroid dysfunction among Type 2 diabetic Saudi patients. Clinical hypothyroidism for patients receiving thyroxin treatment, and Subclinical hypothyroidism when TSH > 5.0 mIU/L with normal T4 (10.55–25.74 pmol/L), while overt hypothyroidism when TSH > 5.0 mIU/L with low T4 <10.55 pmol/L. Subclinical hyperthyroidism when TSH < 0.5 mIU/L with normal T4 (10.55–25.74 pmol/L) and overt hyperthyroidism when TSH < 0.5 mIU/L with high T4 >25.74 pmol/L.

Figure 1 shows the prevalence of different types of thyroid dysfunction among the studied population, where the total prevalence of hypothyroidism was 25.3% and 3.2% for hyperthyroidism. The prevalence of different types of hypothyroidism includes clinical cases (15.3%), sub-clinical (9.5%), and overt hypothyroidism (0.5%). The prevalence of sub-clinical hyperthyroidism in the studied sample was 2.7% and 0.5% for overt hyperthyroidism.

**Figure 2:**
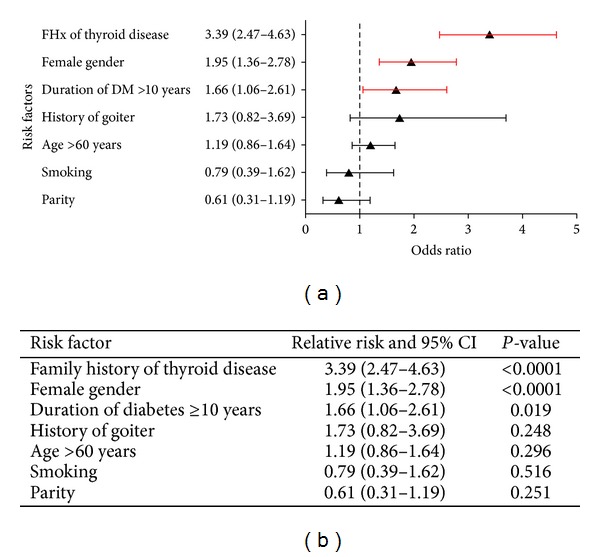
Relative risks for thyroid dysfunction among Type 2 Saudi diabetic patients; (a) forest plot, (b) table of values.

The relative risk and 95% CI for different risk factors for thyroid dysfunction, namely, hypo- or hyperthyroidism for Saudi Type 2 diabetic patients, are shown in Figure 2. Positive family history for thyroid diseases the strongest risk factor with a relative risk (RR) of 3.39 (95% CI, 2.47–4.63) with *P* value < 0.0001, followed by female gender with RR of 1.95 (95% CI, 1.36–2.78) with *P* value < 0.0001. A duration of diabetes of more than 10 years has a relative risk of 1.66 (95% CI, 1.06–2.61) and *P* value = 0.019. Other risk factors, namely, history of goiter and age of more than 60 years, have a relative risk of 1.73 with 95% CI (0.82–3.69) and 1.19 with 95% CI (0.86–1.64) respectively, but both were nonsignificant, with *P* value = 0.248 and 0.296, respectively. Smoking and parity showed low nonsignificant relative risk of 0.79 (0.39–1.62), and 0.61 (0.31–1.19) with *P* value = 0.516 and = 0.251, respectively.

## 5. Discussion

This study has demonstrated that thyroid dysfunction affects more than one quarter of Saudi Type 2 diabetic patients, which is more than that has been reported by Akbar et al. of 16% in 2006 [7] although this study has a bigger sample and older cohort age. In this study, we report the highest prevalence of thyroid dysfunction in Type 2 diabetic patients when compared with other communities, shown by the Scotland study to be 13.4% among both Type 1 diatebes and Type 2 diabetes [6] or by the Jordanian study, where it was 12.5% among Type 2 diabetes [8]. This could be explained by the high prevalence of latent autoimmune diabetes of adult (LDA) in Saudi Type 2 diabetics reaching 26% [7] and in the current study, those patients are not excluded. When comparing our findings of different types of thyroid dysfunction for similar Type 2 diabetic patients' cohort from Spain published by Díez et al. in 2011 [10], we had identical findings for the prevalence of both total and subclinical hypothyroidism cases (25.3% versus 25.8% and 9.5% versus 10.7% resp.). The prevalence of clinical cases of hypothyroidism was 15.3% in our subjects, while overt hypothyroidism in Spain was 15.1%. We had a lower prevalence of total hyperthyroid cases when compared with Spain study (3.2% versus 6.6%) but not for subjects with subclinical hyperthyroidism. Overt hyperthyroid cases were higher in the Spain study when compared with our study (3.5% versus 0.5%).

Diabetic patients with a positive family history of thyroid disease had a higher chance of developing thyroid dysfunction, while the family history of diabetes did not increase the risk for thyroid dysfunction which is the same observation in the United Kingdom DNA collection for Graves' disease and Hashimoto's thyroiditis study [17]. Among Saudi Type 2 diabetic patients of more than 25 years of age, positive family history of thyroid disease is the most prominent risk factor for thyroid dysfunction, as is also shown in Caucasians [32].

As shown in many ethnic groups [8, 20], Saudi diabetic patients with thyroid dysfunction had a significant predominance of female gender. Diabetes duration of more than 10 years in our cohort has been shown to be an important risk factor, which is not the case in the studies of different ethnic groups like Spanish and Chinese population [10, 24]. This may explained by the fact that our cohort had a marked longer duration of 7.3 years versus 9.6 years in Spanish and 8.3 years in Chinese.

We did not find history of goiter to be a significant risk factor for thyroid dysfunction in this retrospective study which was similar to what has been reported by Díez et al. in a more representative prospective study [10], although history of goiter is recognized as a risk factor for thyroid dysfunction in general population [33]. Our results have denied that age is a significant risk factor for thyroid dysfunction, as with what has been reported by different studies [10, 34]. While smoking has been identified as a risk factor for thyroid dysfunction in the general population, especially when smoking is highly prevalent [35], no studies have investigated the impact of smoking as a risk among the diabetic population. We report here that smoking has no effect on thyroid dysfunction among Type 2 diabetic patients. This finding in Type 2 diabetic patients is reported for the first time and has to be taken with special caution, since smoking has very low prevalence among Saudi females for cultural reasons [36].

Parity has been reported in many studies to be a risk factor for thyroid dysfunction in the general population [19, 26], but we did not find this to be the case among Saudi Type 2 diabetic females, although diabetic pregnant women have an increased risk of developing postpartum thyroiditis [27].

Although thyroid autoimmunity is strong risk factors for thyroid dysfunction among diabetic patients, the current retrospective study lacks thyroid antibody data.

We conclude that more than one quarter of Saudi Type 2 diabetic patients of more than 25 years of age are affected by different types of thyroid dysfunction, whereby the majority are hypothyroid cases. Our findings regarding the prevalence of different thyroid dysfunction among Type 2 Saudi diabetic population are higher than those that have been reported by most of studies conducted in different ethnic groups. We have found that family history of thyroid disease, female gender, and duration of diabetes for more than 10 years are significant risk factors for different thyroid dysfunctions.

Based on a high prevalence of thyroid dysfunction among Saudi Type 2 diabetic patients, routine screening for thyroid dysfunction is highly recommended in Saudi diabetic population.

## Figures and Tables

**Table 1:** Baseline characteristics of study sample for all subjects with or without thyroid dysfunction among Type 2 diabetic patients aged >25 years.

	All samples *n* = 411	Diabetic subjects with thyroid dysfunction *n* = 117	Diabetic subjects without thyroid dysfunction *n* = 294	*P* value
Age (years)*	59.0 ± 10.8	59.3 ± 9.9	58.9 ± 11.3	0.732
Female gender^†^	52.3	68.6	46.6	<0.0001
Duration of diabetes in years*	15.8 ± 8.6	17.3 ± 9.0	15.1 ± 8.6	0.032
Family history of diabetes^†^	86.3	84.2	87.1	0.487
Family history of thyroid disease^†^	5.4	14.7	1.03	<0.0001
Positive smoking history^†^	17.4	14.3	18.2	0.516
Weight in kilogram (Kg)*	80.1 ± 15.4	78.6 ± 15.9	80.6 ± 15.2	0.268
Height (cm)*	159.9 ± 9.2	157.3 ± 8.2	161.2 ± 9.5	<0.0001
BMI (kg/m^2^)*	31.5 ± 6.1	32.0 ± 6.2	31.2 ± 5.8	0.209
Systolic blood pressure (mmHg)*	134.8 ± 16.5	134.6 ± 16.9	134.5 ± 16.2	0.926
Diastolic blood pressure (mmHg)*	74.1 ± 9.6	73.1 ± 8.3	74.4 ± 10.0	0.198
Presence of goiter^†^	2.8	4.8	2.1	0.248
HbA1c (%)*	8.5 ± 1.6	8.5 ± 1.5	8.5 ± 1.6	0.943
Fasting blood sugar (mmol/L)*	8.7 ± 2.9	8.6 ± 3.0	8.6 ± 2.8	0.904
Triglycerides (mmol/L)*	1.5 ± 0.78	1.7 ± 0.80	1.5 ± 0.82	0.110
Total cholesterol (mmol/L)*	4.2 ± 0.78	4.3 ± 0.96	4.2 ± 0.68	0.429
HDL (mmol/L)*	1.2 ± 0.33	1.3 ± 0.38	1.2 ± 0.31	0.190
LDL (mmol/L)*	2.3 ± 0.69	2.2 ± 0.81	2.3 ± 0.62	0.427
FT3 (pmol/L)*	4.6 ± 1.4	4.5 ± 2.03	4.7 ± 0.84	0.552
FT4 (pmol/L)*	15.9 ± 3.5	16.6 ± 4.34	15.6 ± 2.7	0.048
TSH (mIU/L)*	3.4 ± 2.9	4.7 ± 4.2	2.6 ± 1.2	<0.0001

*Data presented as mean ± SD, ^†^Data presented as (%), and *P* value is calculated between diabetic subjects with and without thyroid dysfunction.
